# The Short Warwick-Edinburgh Mental Well-being Scale (SWEMWBS): an Italian validation using confirmatory factor analysis and Rasch analysis

**DOI:** 10.1186/s40359-024-02177-8

**Published:** 2024-11-21

**Authors:** Paolo Soraci, Nadia Bevan, Mark D. Griffiths, Renato Pisanti, Rocco Servidio, Ambra Ferrari, Carla Di Bernardo, Amir H. Pakpour

**Affiliations:** 1 Department of Economic, Psychological and Communication Sciences, Niccolò Cusano University, Via Don Carlo Gnocchi 3, Rome, Italy; 2 School of Social Sciences, Faculty of Arts, Monash University, Clayton, VIC Australia; 3 International Gaming Research Unit, Psychology Department, Nottingham Trent University, 50 Shakespeare Street, Nottingham, NG1 4FQ UK; 4 Department of Culture, Education and Society, University of Calabria, Arcavacata di Rende, 87036 Italy; 5 Play Better Associazione, via della Libertà 57/D, Agliana (PT), 51031 Italy; 6 Associazione Psicologi Tecnici Sportivi, Rome, Italy; 7 Department of Nursing, School of Health and Welfare, Jönköping University, Jönköping, Sweden

**Keywords:** Confirmatory factor analysis, Italy, Rasch analysis, Mental health, Short Warwick-Edinburgh Mental Well-being Scale, Well-being

## Abstract

The present study examined the psychometric properties of the Short Warwick-Edinburgh Mental Well-being Scale (SWEMWBS). A total of 742 Italian participants (84.6% female), with a mean age of 33.08 years (SD = 12.44) took part in a cross-sectional survey study. Confirmatory factor analysis and Rasch analysis were used to examine the psychometric properties. The CFA showed that the SWEMWBS had a unidimensional structure with robust psychometric properties and showed good internal consistencies (Cronbach’s alpha 0.88; McDonald’s omega 0.91). Overall, most items showed no substantial differential item functioning in the Rasch analysis, except for Item 7, indicating that female participants reported more difficulty with this item than the male participants. The SWEMWBS was positively associated with life satisfaction and negativity associated with general psychological distress (i.e., anxiety, stress, and depression). In sum, the SWEMWBS showed robust psychometric properties capable of assessing positive aspects of mental health and well-being among Italian-speaking adults.

**Supplementary Information:**

The online version contains Supplementary Material available at 10.1186/s40359-024-02177-8.

## Introduction

The prevalence of poor mental well-being among individuals has increased across European regions [1]. A recent report highlighted that more than 165 million Europeans are affected by mental health disorders, notably anxiety, stress, and depression [2]. Poor mental well-being can have several negative effects on an individual’s life [3]. For example, some research suggests that poor mental health influences physical health, and in turn, this can contribute to poorer physical health, including conditions such as heart disease, some cancers, gastrointestinal disorders, and an impaired immune system [4, 5]. Relationship difficulties can also be affected by poor mental well-being because they can negatively affect interpersonal relationships, leading to conflict, social isolation, and difficulty forming and maintaining meaningful bonds with others [6].

Poor mental well-being can also affect the ability to concentrate, learn, and remember, thereby impairing educational and/or occupational performance (e.g [7]). Moreover, it can negatively affect motivation, energy, and productivity in the workplace [8]. Poor mental well-being may also increase the risk of developing addictions to substances or behaviors, such as drinking alcohol, drug use, or gambling, when they are used as a coping mechanism. Additionally, individuals with poor mental well-being are more likely to experience depression, anxiety, stress, self-harming behavior, and suicidal ideation (increasing the risk of suicide) [9]. The evidence shows that it is essential to take preventive and supportive measures to promote and protect mental health and well-being (e.g [10]).

Mental health and well-being are complex and typically comprise the subjective perception of an individual’s overall welfare and general satisfaction with life, encompassing emotional, psychological, and social aspects [11]. Theoretical perspectives conceptualize mental health and well-being as influenced by both hedonic and eudemonic elements. The former relates to the subjective experience of happiness and fulfillment in life. Eudemonic elements relate to the individual’s psychological health, including their sense of purpose and hopefulness for personal growth [12]. Over the past decade, researchers, policymakers, and service providers have increasingly focused on improving their understanding of the assessment and utilization of mental well-being [10].

Several psychometric instruments have been developed to assess mental health effectively (e.g [7]). However, many studies and tools have focused on a single aspect (for example, some focus more on the hedonic aspect and others on the eudemonic aspect, see [13] for a review). Considering the importance of mental health and well-being and its present theoretical framework, a valid and reliable scale encompassing both hedonic and eudemonic factors is necessary to ensure accurate assessment. An instrument that can address both dimensions will likely have a structural and theoretical advantage over others. Among these, the Warwick-Edinburgh Mental Well-being Scale (WEMWBS) [14] has demonstrated robust psychometric properties, proving its validity and reliability across different cultures and populations, including primary care, community, and employee participant samples [15].

The WEMWBS is a 14-item self-report scale that assesses two main aspects of mental health (i.e., the hedonic and eudemonic components) investigating factors such as general happiness, life satisfaction, and the ability to manage and cope with stress. Therefore, the WEMWBS effectively covers both emotional and functional well-being dimensions [15]. The WEMWBS was developed in response to a growing need to address the increase in public mental health crises. It mainly aims to facilitate the monitoring of nonclinical communities’ mental health and well-being and assesses the effectiveness of interventions, programs, and strategies to promote mental health among those in the general population. Research has also shown that the scale is sensitive to changes resulting from various wellness promotion initiatives [16].

Through qualitative methods, the scale’s development primarily involved mental health service users, who focused on understanding the nature of mental well-being while verifying the apparent validity of the scale [10]. Positive results concerning the validity of the WEMWBS have been confirmed among several subgroups, including healthy adolescents and clinical samples (e.g [17, 18]). Moreover, other validation studies have shown that the WEMWBS is simple to use and provides a reliable assessment of mental well-being [16]. Consequently, the scale has been translated into more than 20 languages, including Urdu, Arabic, Japanese, Chinese, Swedish, Italian, Dutch, German, French, and Spanish. (e.g [18–22]). 

Despite various attempts to confirm the unidimensional structure of the WEMWBS [14], many authors find its factorial configuration controversial (e.g [23, 24]). On one hand, several studies exploring the 14-item structure of the WEMWBS support the unifactorial model (e.g [19, 25–27]). On the other hand, others accept this structure with adjustments, such as including covariances between error terms for specific items (e.g [28, 29]). Other studies reject the unifactorial model in favor of two-factor (e.g [30]), three-factor [31], or even bifactorial models (e.g [24]). Moreover, the Italian version of the WEMWBS [23] comprises 12 items rather than 14 items. According to the extant literature, there is no consensus on the 14-item factorial structure of the WEMWBS.

This lack of agreement emphasizes the need to develop an abbreviated version to eliminate item redundancy since a deviation from the unifactorial model is incongruent with the multidimensional theoretical framework of well-being [10]. Consequently, the Short Warwick-Edinburgh Mental Well-being Scale (SWEMWBS) was then developed through Rasch analysis, demonstrating superior psychometric properties compared to the full 14-item version [32]. Moreover, the SWEMWBS offers the additional advantage of being shorter, therefore easing the cognitive load on participants.

Since its development, the SWEMWBS has been extensively explored in at least 12 published psychometric studies conducted in at least six languages worldwide (but not in Italian), with results consistently favoring the validity of its psychometric characteristics over those of the full version (i.e., unidimensional structure, e.g [28, 33–35]). Consequently, experts now highly recommend the SWEMWBS rather than the WEMWBS as a psychometric tool for assessing mental well-being [10]. Such findings could inform intervention development, support the utilization of the SWEMWBS for assessing mental well-being, and promote strategies to enhance mental well-being among Italy’s population.

The present study addressed this gap by examining whether the good psychometric properties established in other languages would be confirmed in an Italian version. Such validation would provide practitioners with a reliable and concise tool for assessing mental well-being in Italy, supporting both public health interventions and mental health research. In addition, the present study provides specific data on how the SWEMWBS could support mental well-being interventions in Italy, contributing to the standardization and practical application of a brief and reliable tool for assessing mental well-being among individuals the Italian population.

## The present study

The SWEMWBS could be a useful instrument for assessing mental well-being among individuals in Italian population studies. However, its use in the Italian context has not yet been validated. Validation could ensure the relevance and accuracy of the scale in an Italian context. If the validity of the SWEMWBS is established in Italy, it could have significant benefits, including (i) providing a reliable and brief tool for assessing mental well-being in population-based studies, meeting the current high demand for such assessments; (ii) providing mental health promotion practitioners (e.g., physicians, psychologists) with a practical assessment tool for mental well-being, enabling them to assess the effectiveness of their programs; and (iii) providing scientific researchers with the means to study the distribution and predictors of mental well-being, thereby informing national and international mental health policies.

Therefore, the main aim of the present study was to examine the psychometric properties and validate the SWEMWBS among an Italian population sample, hypothesizing that the SWEMWBS (i) has a unidimensional factorial structure with adequate reliability (H_1_), (ii) is positively associated with the WEMWBS and life satisfaction (H_2_), and (iii) is negatively associated with general distress (anxiety, stress, and depression) (H_3_).

## Methods

### Participants and procedure

Participants were recruited via various online channels and social media communities in Italy, such as *Facebook*,* WhatsApp*,* Telegram*, and *Instagram*, through a link advertising a survey hosted on *Google Forms* during a 30-day time period (February-March 2024). The research team disseminated the link, inviting individuals to participate voluntarily and anonymously. No incentive was offered to the participants. The study eligibility criterion for participants was that they were at least 18 years of age and spoke the Italian language. In total, 742 participants completed the questionnaire, most of whom were female (*n* = 641, 84.6%), with an average age of 33.08 years (ranging from 18 to 81 years; SD = 12.44). Approximately one-third of the participants were married (*n* = 244, 32.2%). Regarding educational qualifications, most of the participants had a high school diploma (*n* = 449, 59.2%), followed by a bachelor’s degree (*n* = 164, 21.6%). More than half of the participants were both students and workers (*n* = 436, 57.5%) (see Table S1 to Table S3, Supplementary Materials, for details). Missing data were below the advised thresholds (< 5%) and were missing completely at random (Little, 1988). The pairwise technique controlled the handling of any missing data.

### Measures

#### Demographics

The online survey asked questions about the participants’ demographics, including their gender, age, level of education, and occupation.

#### Depression Anxiety Stress Scale-21 (DASS-21)

The 21-item DASS-21 [36, 37] was used to assess psychological distress. Participants rate items using a four-point scale from 0 (*not at all*) to 3 (*very much*) across three domains: depression (e.g., experiencing a lack of excitement), anxiety (e.g., approaching a panic attack), and stress (e.g., finding it difficult to relax). Scores for each domain range from 0 to 21, with the total score ranging from 0 to 63, representing overall psychological distress calculated by summing of the three domain scores. Higher scores on individual domains indicate elevated levels of depression, anxiety, and stress. The Cronbach’s alphas (depression α = 0.88, anxiety α = 0.89, and stress α = 0.86) and McDonald’s omegas (depression ω = 0.86, anxiety ω = 0.88, and stress ω = 0.88) in the present study were very good.

#### Warwick-Edinburgh Mental Well-Being Scale (WEMWBS)

The 12-item Italian Warwick-Edinburgh Mental Well-being Scale (WEMWBS [14, 23]), was used to assess an individual’s mental well-being. Each item is rated on a five-point Likert scale ranging from 1 (*None of the time*) to 5 (*All of the time*). Examples of items include “*I’ve been feeling optimistic about the future*” and “*I’ve been feeling cheerful*”. The total score is calculated by summing the scores of individual items. The total score ranges from 12 to 60. A higher score indicates greater mental well-being. The Cronbach’s alpha (α = 0.87) and McDonald’s omega (ω = 0.88) in the present study were very good.

#### Short Warwick-Edinburgh Mental Well-being Scale (SWEMWBS)

The SWEMEBS is a short version of the WEMWBS, comprising seven positively worded items rated on a seven-point scale from 1 (*none of the time*) to 5 (*all of the time*). The total score is calculated by summing the scores of individual items. The total score ranges from 7 to 35. A higher score indicates greater mental well-being. The participants were asked to respond to the Italian 12-item scale, and the responses were then used to calculate scores for the seven-item scale (by using Items 1, 2, 3, 7, 8, 9, 11, see the Appendix in the Supplementary Materials) without requiring participants to respond to those items separately. The psychometric properties are reported in the ‘Results’ section.

#### Satisfaction with Life Scale (SWLS)

The five-item SWLS [38] was used to assess life satisfaction. The items are rated on a seven-point scale ranging from 1 (*totally disagree*) to 7 (*fully agree*). An example item is “*Most aspects of my life are as I want them to be*”. The total score ranges from 7 to 35. A higher score indicates greater satisfaction with life. The Cronbach’s alpha (α = 0.85) and McDonald’s omega (ω = 0.86) in the present study were very good.

### Ethical approval

The study was conducted according to the Declaration of Helsinki for medical research involving human participants and was approved by the Ethical Committee of Niccolò Cusano University, Rome (January 26, 2024). All participants gave their informed consent to participate in the study. The identities of the participants were anonymous, and the data were stored in an encrypted online archive accessible only to the authors of the present study.

### Data analysis

The demographic characteristics of the participants were analyzed using descriptive analyses (e.g., means, medians, frequencies, percentages).

#### Validity: factor structure

The factorial structure of the SWEMWBS was assessed using confirmatory factor analysis (CFA). To estimate the CFA model, the diagonally weighted least square estimator was used. To assess the goodness of fit of the model, several appropriate fit indices were used [39, 40] including the comparative fit index (CFI > 0.95), the square error of approximation (RMSEA < 0.08), the Tucker Lewis index (TLI > 0.95), and the standardized root mean squared residual (SRMR < 0.06). Because the data were non-normally distributed (i.e., significant Shapiro–Wilk *p* < 0.001 for the SWEMWBS items; see Table S4 and S5 [Supplementary Materials] for details), the diagonally weighted least squares (DWLS) estimator was used to conduct confirmatory factor analyses (CFAs).

#### Assessment of convergent and discriminant validity

Moreover, all items should achieve acceptable saturation (λij ≥ 0.50; [41]). Both convergent and discriminant validity were assessed through the “measureQ” function following the recommendations of Cheung et al. [41], outlining the construction of a model, and containing the main measures used (i.e., SWEMWBS, SWLS, stress, anxiety, and depression [DASS-21]) (see model code in Supplementary Materials). Through the “measureQ” function and the construction of the related model it is possible to examine (in detail) the standardized factor loadings, average variance extracted, cross-loaded indicators, and heterotrait-monotrait in one single feature. It also provides information on whether the assessed constructs are appropriate (i.e., whether all indicators are related to the constructs they are intended to assess and unrelated to the other constructs in the model), through the use of the main model fits (i.e., RMSEA, CFI, SRMR). When the overall model fit indices demonstrate that the hypothesized measurement model aligns well with the data [41], it satisfies the core prerequisite for assessing reliability, as well as convergent and discriminant validity.

Furthermore, Cheung et al. [41] suggest the following guidelines. For convergent validity, the criteria included the following: (i) construct reliability (CR) values equal to or exceeding 0.7, (ii) all standardized factor loadings (λ) being 0.5 or higher, and (iii) average variance extracted (AVE) values being 0.5 or higher. For discriminant validity, the following criteria were used: absence of indicator cross-loads on other constructs, maximum shared variance (MSV) < AVE, and heterotrait–monotrait ratio of correlations (HTMT) approach, with values < 0.85 indicating good discriminant validity [41]. In addition, convergent validity was further analyzed by examining the correlations between the Italian version of SWEMWBS and the constructs theoretically associated with it (i.e., life satisfaction, stress, anxiety, depression) [38, 39]. Parsimony indices, in particular the expected cross-validation index (ECVI), were used in the analysis. However, smaller values suggest greater parsimony and model fit [42].

#### Factor structure assessed by Rasch analysis

Rasch analysis using a partial credit model was conducted to assess the unidimensionality of the Italian SWEMWBS. To measure item fit, the infit and outfit mean square (MnSq) were estimated with values between 0.6 and 1.4, which are considered acceptable fits [43]. To assess the unidimensional structure of the SWEMWBS, a principal component analysis (PCA) of the residuals was conducted on the Rasch model. To confirm unidimensionality, at least 50% of the raw variance should be explained by the measure, and the first contact of the residual should have an eigenvalue < 2.0 [43]. Local dependency was assessed by calculating the standardized residual correlations between two items with values higher than 0.30 indicating local dependency. Differential item functioning (DIF) was used to examine the measurement invariance of SWEMWBS items across gender groups, with DIF > 1.0 logit indicating substantial DIF [44].

#### Reliability

The internal consistency of SWEMWBS was evaluated using Cronbach’s α and McDonald’s ω, with values greater than 0.70 considered acceptable. Additionally, composite reliabilities exceeding 0.6 and corrected item-total correlations higher than 0.40 were deemed satisfactory [41]. Finally, Spearman’s rho correlation coefficients were calculated to examine the relationships between the SWEMEBS scores and other measures.

#### Analysis software

The analysis utilized SPSS Statistics v.27 for descriptive statistics [45], R Studio for convergent and discriminant validity (“measureQ” package) [46], and JASP version 0.18.3 for correlation analysis [47].

## Results

### Descriptive statistics of the main measures used

With regards to the measures used, the results were as follows: the mean score on the SWEMWBS was 24.77 out of 35 (SD = 5.45); on the WEMWBS it was 42.06 out of 60 (SD = 9.48); and on the SWLS it was 22.58 out of 35 (SD = 6.83). In addition, the mean score on the DASS-21 stress subscale was 11.140 out of 21 (SD = 5.40); on the DASS-21 anxiety subscale it was 7.08 out of 21 (SD = 5.75); and on the DASS-21 depression subscale it was 7.94 out of 21 (SD = 5.99). Finally, the mean global score on the DASS-21 was 26.10 out of 63 (SD = 15.72). For details on the descriptive properties of the main measures used, see Tables S1, S2 and S3 in the Supplementary Materials.

### Confirmatory factor analysis (CFA)

The results of the CFA indicated a good factorial structure of the SWEMWBS: chi-square test (χ^2^) = 24.03 (*df* = 14, *n* = 742; with *χ*^2^/df = 1.71); *p* > 0.05, CFI = 0.99, TLI = 0.99, RMSEA = 0.018, 90% CI (0.001, 0.042), *p* = 0.990 (*p* > 0.05), SRMR = 0.037, GFI = 0.998. All factor loadings exhibited high and statistically significant values for all items (min = 0.66, max = 0.84; i.e., λij ≥ 0.50; see Table 1 for details). The item-total correlation was also satisfactory (min = 0.67, max = 0.82). Finally, the ECVI = 0.069.

**Table 1:** Factor loadings of items in the Short Warwick-Edinburgh Mental Well-being Scale

						95% CI			
Factor	Indicator	Estimate	Std. Error	z value	*p*	Lower	Upper	Std. Est. (all)	
SWEMEBS	ITEM 1	0.837	0.025	32.893	< 0.001	0.787	0.887	0.804	
	ITEM 2	0.684	0.024	28.489	< 0.001	0.637	0.731	0.662	
	ITEM 3	0.722	0.023	31.014	< 0.001	0.676	0.767	0.708	
	ITEM 4	0.940	0.027	34.254	< 0.001	0.886	0.994	0.793	
	ITEM 5	0.823	0.025	32.836	< 0.001	0.774	0.872	0.837	
	ITEM 6	0.709	0.024	29.230	< 0.001	0.662	0.757	0.734	
	ITEM 7	0.930	0.027	34.561	< 0.001	0.877	0.983	0.839	

Std. Est. (all) = Estimate standardized factor loading

Subsequently, the model of the SWEMWBS was compared with the original Italian 12-item (WEMWBS) version, to test whether the shorter seven-item version had comparable/better psychometric properties than the full 12-item version. The results of the CFA of the 12-item Italian version (WEMWBS) were: chi-square test (χ^2^) = 97.39 (*df* = 54, *n* = 742; with χ^2^/df = 1.80); *p* < 0.01, CFI = 0.99, TLI = 0.99, RMSEA = 0.030, 90% CI (0.02, 0.04), *p* = 0.99 (*p* > 0.05), SRMR = 0.05, GFI = 0.99, ECVI = 0.209. All factor loadings exhibited high and statistically significant values for all items (min = 0.51, max = 0.83; i.e., λij ≥ 0.50, see Table S11 in Supplementary Materials).

The results showed that the seven-item short version (SWEMWBS) had similar goodness-of-fit indices to the 12-item version (WEMWBS), although the ECVI was lower, and therefore more parsimonious, in the seven-item model. For the subsequent analysis, the seven-item version (SWEMWBS) was used (see Table 2 for details).

**Table 2:** CFA comparison of the Short Warwick-Edinburgh Mental Well-being Scale and Warwick-Edinburgh Mental Well-being Scale

	χ^2^	χ^2^/df	CFI	TLI	RMSA	SRMR	ECVI
SWEMWBS	24.03	1.71	0.99	0.99	0.018	0.037	0.069
WEMWBS	97.39	1.80	0.99	0.99	0.030	0.050	0.209

*χ*^2^ chi-square, *df* degrees of freedom, *ECVI* Expected cross-validation index, *CFI* Comparative fit index, *TLI* Tucker–Lewis’s index, *RMSEA* Root mean square error of approximation, *SRMR* Standardized root-mean-square residual

### Rasch analysis

The results of the Rasch analysis are shown in Table 3. The item separation reliability and index were 0.99 and 8.32, respectively. The Pearson separation reliability and index were 0.88 and 2.69, respectively. All the values were within the acceptable range (the infit MnSq ranged from 0.70 to 1.37, and the outfit MnSq ranged from 0.68 to 1.32; although SWEMWBS’s outfit MnSq of 0.68 was slightly below the accepted range of 0.7, indicating a minor overfit). Item 2 was the easiest item, while Item 3 was the most difficult item in the Rasch model. The unidimensional structure of the SWEMWBS was confirmed by the PCA of the residuals, with 62% of the raw variance explained by the measure (Eigenvalue = 11.74). The first contrast had an eigenvalue of 1.75, which explained 9.3% of the variance. Local dependency was not observed, as there were no correlations between items higher than 0.3. DIF was assessed across sex groups, and the results are shown in Table 3. Overall, most items showed no substantial DIF. However, Item 7 showed substantial DIF, indicating that female participants reported more difficulty with this item than male participants.

**Table 3:** Rasch analysis results for Short Warwick-Edinburgh Mental Well-being scale scale items

Item No.	Rasch Analyses				
	**Infit MnSq**	**Outfit MnSq**	Difficulty	Discrimination	DIF contrast across gender^a^
SWEMWBS1	0.88	0.89	-0.20	1.12	0
SWEMWBS2	1.37	1.32	-0.60	0.68	-0.47
SWEMWBS3	1.07	1.10	0.94	0.89	-0.60
SWEMWBS4	1.09	1.09	0.46	0.88	0.15
SWEMWBS5	0.70	0.68	-0.28	1.30	0.04
SWEMWBS6	1.00	0.96	-0.49	1.01	-0.55
SWEMWBS7	0.84	0.85	0.17	1.18	1.63

^a^Gender: females - males

### Reliability

To test the reliability of the SWEMWBS, different reliability metrics (i.e., internal consistency), such as Cronbach’s alpha, McDonald’s omega, and composite reliability (CR), were used and analyzed. Cronbach’s alpha was 0.886 and could not be improved by removing any items. Similarly, McDonald’s omega had a value of 0.886. The CR was 0.91. Given these results, the reliability of the SWEMWBS was considered very good. See Table 4 for details.

**Table 4:** Reliability of the Short Warwick-Edinburgh Mental Well-being Scale

Estimate	Ω	α	AVC
Point estimate	0.911	0.877	0.565
95% CI lower bound	0.871	0.873	0.528
95% CI upper bound	0.902	0.898	0.603

*AVC* Average interitem correlation. α = Cronbach’s α. ω = McDonald’s ω

### Convergent and discriminant validity

To investigate convergent and discriminant validity, a model was constructed (using R’s “measureQ” function, see model code in Supplementary Materials), following the recommendations of Cheung et al. [41], which included the present study’s main scales and subscales (i.e., SWEMWBS, SWLS, stress, anxiety, and depression [DASS-21]). The adaptation of the overall model was good: χ^2^ = 1434.07 *(df* = 485), RMSEA = 0.053, 90% CI (0.050, 0.056), *p* = 0.56), CFI = 0.93, and SRMR = 0.044. All items, as shown in Table S8 (Supplementary Materials), had a standardized factorial load not significantly lower than 0.7. The AVE (0.52) of the SWEMWBS factor was not significantly lower than 0.5, which raised no concern. The construct reliability was also satisfactory with a value of 0.88. Therefore, convergent validity was confirmed. Discriminant validity was also examined. First, the measurement model had no secondary loadings and fitted the data well, and the MSV was < AVE (for details regarding convergent and discriminant validity results, see Table S7, S8 and S9, Supplementary Materials). Moreover, the HTMT correlation matrix was assessed (Table S10, Supplementary Materials). No value was significantly greater than 0.85. Therefore, the discriminant validity was also confirmed.

### Correlation analysis

The correlation analysis showed that the SWEMWBS score was significantly associated with the WEMWBS score and life satisfaction score (SWLS) and was negatively and significantly correlated with general psychological distress (total DASS-21 score, and subscale scores [i.e., anxiety, stress, and depression]). All the correlations were significant (*p* < 0.001). See Table S6 (Supplementary Materials) for details. These results reinforce the convergent and discriminant validity of the SWEMWBS.

## Discussion

The aim of the present study was to evaluate the psychometric properties of the SWEMWBS among the Italian individuals in the general population. Although there is an Italian version of the full 14-item scale of the WEMWBS, to the best of the present authors’ knowledge, no previous study has ever assessed the shortened seven-item version of the SWEMWBS in the Italian language. Additionally, the full Italian version of the WEMWBS recommends the omission of two items to improve the validity and reliability of the scale, making it an effective 12-item scale. One important limitation of omitting these items is the decreased capacity for international comparisons. Therefore, a concise measure of positive mental wellbeing is needed in Italian culture to facilitate international comparisons.

The results of the present study generally showed that the SWEMWBS had very good reliability and a unidimensional factor structure. As in previous studies [10, 14, 21, 48–50], it is not surprising that the Italian version of the SWEMWBS demonstrated high internal consistency. All seven items assessed the same construct with an adequate sample size, and strong intercorrelations between items. Moreover, there was a very high correlation coefficient between the full and short versions of the WEMWBS (*r* = 0.972) suggesting that the short version (SWEMWBS) can effectively substitute for the long version without compromising the theoretical framework. Previous studies have also reported strong correlations between the full and short versions of the WEMWBS [17, 51, 52].

Consistent with previous studies, the factor structure of the Italian SWEMWBS was confirmed through CFA and Rasch analysis, suggesting the calculation of a meaningful total score by summing individual item scores [10, 17, 50, 51, 53, 54]. Fit indices were also adequate. However, conceptually, mental well-being is considered a multidimensional concept that covers at least two aspects of mental well-being (i.e., subjective experience of happiness and life satisfaction [hedonic aspects], and an individual’s psychological function, sense of purpose, and personal growth [eudemonic aspects]). Although the full 14-item version of the WEMWBS includes both hedonic and eudemonic aspects of mental well-being, the short version of this scale includes more eudemonic aspects. Therefore, the total score of the SWEMWBS is arguably more representative of the overall sense of fulfillment, meaning, and flourishing in life.

The correlations in the present study showed that SWEMWBS was negatively associated with anxiety, stress, and depression, and positively associated with life satisfaction. These significant correlations show that mental well-being could be a distinct concept. Consistent with previous research [55], psychological problems (i.e., stress, anxiety, depression, and general psychological distress) can reduce life satisfaction and diminish mental well-being. In the present study, it was found that the SWEMWBS score was negatively associated with anxiety, stress, and depression, and positively associated with life satisfaction. However, the relationship between psychological problems and mental well-being is complex and cannot be fully captured through a cross-sectional study. Longitudinal studies may be better suited to understand these concepts further.

The convergent and discriminant validity of the SWEMWBS was established following the latest psychometric guidelines, providing a robust and in-depth analysis [41]. More specifically, the average variance extracted (AVE) scores for the SWEMWBS unidimensional factor exceeded the ideal threshold of 0.50, indicating that the items within each factor adequately captured the core essence of that particular factor. This indicates robust convergent validity because higher AVE scores imply a stronger correlation among items within the same construct. The present study also showed that the heterotrait–monotrait (HTMT) ratio of correlation values were below the threshold of 0.85, which is within the allowed limits. Moreover, having all standardized factor loadings (λ) of 0.5 or higher, the absence of indicator cross-loads on other constructs, and MSV < AVE indicate adequate convergent and discriminant validity [41].

Rasch analysis of the SWEMWBS showed that there were no significant differences between males and females with six items of the SWEMWBS. However, Item 7 (*“I’ve been able to make up my own mind about things”*) was found to be significantly more difficult for females than for males (DIF = 1.63). This result contrasts with previous studies on the SWEMWBS indicating a gender-neutral role for this scale (e.g [21, 49]). This may be due to several factors, including, for example, (i) the over-representation of the female gender in the present study (over 80% of participants), and (ii) by intrinsic characteristics to the sample that participated in the study.

The validation of the Italian SWEMWBS may be useful for international researchers, such as those wishing to conduct cross-cultural comparisons (e.g., Italy-Spain) or translating it into another language, using the present results as a comparison tool.

### Limitations and future directions

Despite the promising results, the present study has some limitations. First, the study used convenience sampling, which may limit the generalizability and representativeness of the results. Second, participants’ responses may have been influenced by social desirability bias. Third, participants did not have a formal diagnosis of depression, anxiety, or stress disorders. Fourth, an adequate CFA could not be performed on gender invariance, especially since the sample consisted mostly of female participants (over 80%). Finally, the study was a cross-sectional research design, which has inherent limitations. For example, investigating the cause-and-effect relationships of the variables studied was not possible.

Future studies, such as longitudinal research with a more representative sample, should attempt to replicate the results found in the present study in more detail, including the addition of other variables, such as personality factors or coping mechanisms used by individuals to cope with stressful and anxiety-inducing situations. Moreover, future studies should perform CFA invariance by gender. Future studies could also verify the present study’s Rasch analysis results. In addition, for greater robustness, future studies should perform CFA comparison analyses between the models of the 7-item version, the 12-item version, and the 14-item version of the WEMWBS. Finally, other studies should compare the results of the present study with other international results (e.g., a comparison of Italian results with [say] Spanish or Danish results).

## Conclusion

The present study showed that the Italian SWEMWBS has good psychometric properties and is an appropriate instrument for assessing well-being among the Italian adult population. For practical reasons, the short version (which has also been validated elsewhere in other contexts (e.g.,[10]) may be preferred by both researchers and practitioners (as well as by the participants completing it).

## Supplementary Information

Supplementary Material 1.

## Data Availability

Research data are available upon reasonable request to the first author.

## Abbreviations

AVC: Average interitem correlation
CFI: comparative fit index
Df: degrees of freedom
ECVI: expected cross-validation index
RMSEA: root mean square error of approximation
SRMR: standardized root-mean-square residual
Std. Est. (all): estimate standardized factor loading
SWEMWBS: Short Warwick-Edinburgh Mental Well-being Scale
SWL: Satisfaction With Life Scale
TLI: Tucker–Lewis’s index
WEMWBS: Warwick-Edinburgh Mental Well-Being Scale
α: Cronbach’s α
χ^2^: chi-square
ω: McDonald’s ω

## References

[1] Ahmed N, Barnett P, Greenburgh A, Pemovska T, Stefanidou T, Lyons N, et al. Mental health in Europe during the COVID-19 pandemic: a systematic review. Lancet Psychiatry. 2023;10(7):537–56. 10.1016/S2215-0366(23)00113-X. 37321240 10.1016/S2215-0366(23)00113-X PMC10259832

[2] Trautmann S, Rehm J, Wittchen HU. The economic costs of mental disorders: do our societies react appropriately to the burden of mental disorders? EMBO Rep. 2016;17(9):1245–9. 10.15252/embr.201642951. 27491723 10.15252/embr.201642951 PMC5007565

[3] Shah N, Cader M, Andrews B, McCabe R, Stewart-Brown SL. Short Warwick-Edinburgh Mental Well-being Scale (SWEMWBS): performance in a clinical sample in relation to PHQ-9 and GAD-7. Health Qual Life Outcomes. 2021;19(1):260. 10.1186/s12955-021-01882-x. 34819104 10.1186/s12955-021-01882-x PMC8611866

[4] Doherty AM, Gaughran F. The interface of physical and mental health. Soc Psychiatry Psychiatr Epidemiol. 2014;49(5):673–82. 10.1007/s00127-014-0847-7. 24562320 10.1007/s00127-014-0847-7

[5] Nabi H, Kivimaki M, De Vogli R, Marmot MG, Singh-Manoux A, Whitehall IIPCS. Positive and negative affect and risk of coronary heart disease: Whitehall II prospective cohort study. BMJ. 2008;337(7660):a118. 10.1136/bmj.a118. 18595926 10.1136/bmj.a118 PMC2443594

[6] Steptoe A, Shankar A, Demakakos P, Wardle J. Social isolation, loneliness, and all-cause mortality in older men and women. Proc Natl Acad Sci U S A. 2013;110(15):5797–801. 10.1073/pnas.1219686110. 23530191 10.1073/pnas.1219686110 PMC3625264

[7] Taggart F, Stewart-Brown S. A review of questionnaires designed to measure mental wellbeing.https://warwick.ac.uk/fac/sci/med/research/platform/wemwbs/research/validation/frances_taggart_research.pdf (2019). Accessed 19 April 2024.

[8] Contoyannis P, Rice N. The impact of health on wages: evidence from the British Household Panel Survey. Empirical Economics. 2001;26:599–622.

[9] Bradvik L. Suicide risk and mental disorders. Int J Environ Res Public Health. 2018;15(9). 10.3390/ijerph15092028. 30227658 10.3390/ijerph15092028 PMC6165520

[10] Pakpour AH, Eriksson M, Erixon I, Brostrom A, Bengtsson S, Jakobsson M, Huus K. The short Warwick-Edinburgh Mental Well-being scale (SWEMWBS) - a psychometric evaluation of adolescents in Sweden during the COVID-19 pandemic. Heliyon. 2024;10(6):e27620. 10.1016/j.heliyon.2024.e27620. 38510050 10.1016/j.heliyon.2024.e27620 PMC10950601

[11] Promotion of mental. well-being: pursuit of happiness. https://www.who.int/publications/i/item/9789290224228 (2013). Accessed 29 April 2024.

[12] Keyes CL, Annas J. Feeling good and functioning well: distinctive concepts in ancient philosophy and contemporary science. J Posit Psychol. 2009;4(3):197–201.

[13] Linton MJ, Dieppe P, Medina-Lara A. Review of 99 self-report measures for assessing well-being in adults: exploring dimensions of well-being and developments over time. BMJ Open. 2016;6(7):e010641. 10.1136/bmjopen-2015-010641. 27388349 10.1136/bmjopen-2015-010641 PMC4947747

[14] Tennant R, Hiller L, Fishwick R, Platt S, Joseph S, Weich S, et al. The Warwick-Edinburgh Mental Well-being scale (WEMWBS): development and UK validation. Health Qual Life Outcomes. 2007;5:63. 10.1186/1477-7525-5-63. 18042300 10.1186/1477-7525-5-63 PMC2222612

[15] Stewart-Brown S. The Warwick-Edinburgh Mental Well-being scale (WEMWBS): performance in different cultural and geographical groups. In: Keyes CLM, editor. Mental well-Being: International contributions to the study of positive mental health. Dordrecht, Netherlands: Springer; 2013. pp. 133–50.

[16] Stewart-Brown S. Warwick-Edinburgh Mental Wellbeing Scale (WEMWBS). http://www2.warwick.ac.uk/fac/med/research/platform/wemwbs/ (2015). Accessed 29 April 2024.

[17] McKay MT, Andretta JR. Evidence for the psychometric validity, internal consistency and measurement invariance of Warwick Edinburgh Mental Well-Being Scale Scores in Scottish and Irish adolescents. Psychiatry Res. 2017;255:382–6. 10.1016/j.psychres.2017.06.071. 28666244 10.1016/j.psychres.2017.06.071

[18] Smith ORF, Alves DE, Knapstad M, Haug E, Aaro LE. Measuring mental well-being in Norway: validation of the Warwick-Edinburgh Mental Well-being scale (WEMWBS). BMC Psychiatry. 2017;17(1):182. 10.1186/s12888-017-1343-x. 28499368 10.1186/s12888-017-1343-x PMC5427526

[19] Castellvi P, Forero CG, Codony M, Vilagut G, Brugulat P, Medina A, et al. The Spanish version of the Warwick-Edinburgh mental well-being scale (WEMWBS) is valid for use in the general population. Qual Life Res. 2014;23(3):857–68. 10.1007/s11136-013-0513-7. 24005886 10.1007/s11136-013-0513-7

[20] Forero CG, Adroher ND, Stewart-Brown S, Castellvi P, Codony M, Vilagut G, et al. Differential item and test functioning methodology indicated that item response bias was not a substantial cause of country differences in mental well-being. J Clin Epidemiol. 2014;67(12):1364–74. 10.1016/j.jclinepi.2014.06.017. 25150627 10.1016/j.jclinepi.2014.06.017

[21] Haver A, Akerjordet K, Caputi P, Furunes T, Magee C. Measuring mental well-being: a validation of the short Warwick-Edinburgh Mental Well-being scale in Norwegian and Swedish. Scand J Public Health. 2015;43(7):721–7. 10.1177/1403494815588862. 26041133 10.1177/1403494815588862

[22] Taggart F. WEMWBS in other languages. (2015). Accessed 29 April 2024.

[23] Gremigni PS-B. Measuring mental well-being: Italian validation of the Warwick-Edinburgh Mental Well-Being Scale Giornale Italiano. Di Psicologia. 2011;38(2):485–505.

[24] Shannon S, Breslin G, Prentice G, Leavey G. Testing the factor structure of the Warwick-Edinburgh Mental Well-being scale in adolescents: a bi-factor modelling methodology. Psychiatry Res. 2020;293:113393. 10.1016/j.psychres.2020.113393. 32835928 10.1016/j.psychres.2020.113393

[25] Dong A, Chen X, Zhu L, Shi L, Cai Y, Shi B, et al. Translation and validation of a Chinese version of the Warwick-Edinburgh Mental Well-being scale with undergraduate nursing trainees. J Psychiatr Ment Health Nurs. 2016;23(9–10):554–60. 10.1111/jpm.12344. 27860080 10.1111/jpm.12344

[26] Marmara J, Zarate D, Vassallo J, Patten R, Stavropoulos V. Warwick Edinburgh Mental Well-Being Scale (WEMWBS): measurement invariance across genders and item response theory examination. BMC Psychol. 2022;10(1):31. 10.1186/s40359-022-00720-z. 35183262 10.1186/s40359-022-00720-z PMC8857792

[27] Trousselard M, Steiler D, Dutheil F, Claverie D, Canini F, Fenouillet F, et al. Validation of the Warwick-Edinburgh Mental Well-being scale (WEMWBS) in French psychiatric and general populations. Psychiatry Res. 2016;245:282–90. 10.1016/j.psychres.2016.08.050. 27565700 10.1016/j.psychres.2016.08.050

[28] Fung SF. Psychometric evaluation of the Warwick-Edinburgh Mental Well-being scale (WEMWBS) with Chinese university students. Health Qual Life Outcomes. 2019;17(1):46. 10.1186/s12955-019-1113-1. 30871563 10.1186/s12955-019-1113-1 PMC6416978

[29] Perera BPR, Caldera A, Godamunne P, Stewart-Brown S, Wickremasinghe AR, Jayasuriya R. Measuring mental well-being in Sri Lanka: validation of the Warwick Edinburgh Mental Well-Being Scale (WEMWBS) in a Sinhala speaking community. BMC Psychiatry. 2022;22(1):569. 10.1186/s12888-022-04211-8. 35999535 10.1186/s12888-022-04211-8 PMC9400250

[30] Waqas A, Ahmad W, Haddad M, Taggart FM, Muhammad Z, Bukhari MH, et al. Measuring the well-being of health care professionals in the Punjab: a psychometric evaluation of the Warwick-Edinburgh Mental Well-being Scale in a Pakistani population. PeerJ. 2015;3:e1264. 10.7717/peerj.1264. 26557420 10.7717/peerj.1264 PMC4636413

[31] Sarasjarvi KK, Elovainio M, Appelqvist-Schmidlechner K, Solin P, Tamminen N, Therman S. Exploring the structure and psychometric properties of the Warwick-Edinburgh Mental Well-being scale (WEMWBS) in a representative adult population sample. Psychiatry Res. 2023;328:115465. 10.1016/j.psychres.2023.115465. 37708805 10.1016/j.psychres.2023.115465

[32] Stewart-Brown S, Tennant A, Tennant R, Platt S, Parkinson J, Weich S. Internal construct validity of the Warwick-Edinburgh Mental Well-being scale (WEMWBS): a Rasch analysis using data from the Scottish Health Education Population Survey. Health Qual Life Outcomes. 2009;7:15. 10.1186/1477-7525-7-15. 19228398 10.1186/1477-7525-7-15 PMC2669062

[33] Anthony R, Moore G, Page N, Hewitt G, Murphy S, Melendez-Torres GJ. Measurement invariance of the short Warwick-Edinburgh Mental Wellbeing Scale and latent mean differences (SWEMWBS) in young people by current care status. Qual Life Res. 2022;31(1):205–13. 10.1007/s11136-021-02896-0. 34050443 10.1007/s11136-021-02896-0 PMC8800901

[34] Hauch D, Fjorback LO, Juul L. Psychometric properties of the short Warwick-Edinburgh Mental Well-being scale in a sample of Danish schoolchildren. Scand J Public Health. 2023;51(8):1214–21. 10.1177/14034948221110002. 35855556 10.1177/14034948221110002

[35] Melendez-Torres GJ, Hewitt G, Hallingberg B, Anthony R, Collishaw S, Hall J, et al. Measurement invariance properties and external construct validity of the short Warwick-Edinburgh mental wellbeing scale in a large national sample of secondary school students in Wales. Health Qual Life Outcomes. 2019;17(1):139. 10.1186/s12955-019-1204-z. 31412878 10.1186/s12955-019-1204-z PMC6694652

[36] Henry JD, Crawford JR. The short-form version of the Depression anxiety stress scales (DASS-21): construct validity and normative data in a large non-clinical sample. Br J Clin Psychol. 2005;44(Pt 2):227–39. 10.1348/014466505X29657. 16004657 10.1348/014466505X29657

[37] Bottesi G, Ghisi M, Altoe G, Conforti E, Melli G, Sica C. The Italian version of the Depression anxiety stress Scales-21: factor structure and psychometric properties on community and clinical samples. Compr Psychiatry. 2015;60:170–81. 10.1016/j.comppsych.2015.04.005. 25933937 10.1016/j.comppsych.2015.04.005

[38] di Fabio A, Palazzeschi L. The satisfaction with Life Scale (SWLS): Un contributo alla validazione italiana con lavoratori adulti. Counseling: Giornale Italiano Di Ricerca E Applicazioni. 2012;5(2):207–15.

[39] Kline RB. Ethodology in the social sciences. Principles and practice of structural equation modeling. third ed. Guilford Press; 2011.

[40] Kline RB. Principles and practice of structural equation modeling. fourth ed. Guilford Press; 2016.

[41] Cheung GW, Cooper-Thomas HD, Lau RS, Wang LC. Reporting reliability, convergent and discriminant validity with structural equation modeling: a review and best-practice recommendations. Asia Pac J Manage. 2024;41:745–83. 10.1007/s10490-023-09871-y.

[42] Byrne BM. Structural equation modeling with AMOS. Taylor & Francis; 2016.

[43] Williams B, Onsman A, Brown T. A Rasch and factor analysis of a paramedic graduate attribute scale. Eval Health Prof. 2012;35(2):148–68. 10.1177/0163278711407314. 21613243 10.1177/0163278711407314

[44] Canto-Cerdan M, Cacho-Martinez P, Lara-Lacarcel F, Garcia-Munoz A. Rasch analysis for development and reduction of Symptom Questionnaire for visual dysfunctions (SQVD). Sci Rep. 2021;11(1):14855. 10.1038/s41598-021-94166-9. 34290288 10.1038/s41598-021-94166-9 PMC8295373

[45] Corp I. IBM SPSS statistics for Windows (Version 27.0. IBM Corp; 2020.

[46] Team RC. R: A language and environment for statistical computing. https://www.R-project.org/ (2021). Accessed November 6, 2024.

[47] Team J. JASP (Version 0.18.3) https://jasp-stats.org/ (2020). Accessed November 6, 2024.

[48] Hunter SH, Wood S. Positive mental well-being in Australian adolescents: evaluating the Warwick-Edinburgh Mental Well-being Scale. Aust Educ Develop Psychol. 2015;32(2):93–104.

[49] Ng SS, Lo AW, Leung TK, Chan FS, Wong AT, Lam RW, Tsang DK. Translation and validation of the Chinese version of the short Warwick-Edinburgh Mental Well-being scale for patients with mental illness in Hong Kong. East Asian Arch Psychiatry. 2014;24(1):3–9. 24676481

[50] Sun Y, Luk TT, Wang MP, Shen C, Ho SY, Viswanath K, et al. The reliability and validity of the Chinese short Warwick-Edinburgh Mental Well-being Scale in the general population of Hong Kong. Qual Life Res. 2019;28(10):2813–20. 10.1007/s11136-019-02218-5. 31144205 10.1007/s11136-019-02218-5

[51] Koushede V, Lasgaard M, Hinrichsen C, Meilstrup C, Nielsen L, Rayce SB, et al. Measuring mental well-being in Denmark: validation of the original and short version of the Warwick-Edinburgh Mental Well-being scale (WEMWBS and SWEMWBS) and cross-cultural comparison across four European settings. Psychiatry Res. 2019;271:502–9. 10.1016/j.psychres.2018.12.003. 30551082 10.1016/j.psychres.2018.12.003

[52] Ringdal R, Bradley Eilertsen ME, Bjornsen HN, Espnes GA, Moksnes UK. Validation of two versions of the Warwick-Edinburgh Mental Well-being scale among Norwegian adolescents. Scand J Public Health. 2018;46(7):718–25. 10.1177/1403494817735391. 29017402 10.1177/1403494817735391

[53] Bartram DJ, Sinclair JM, Baldwin DS. Further validation of the Warwick-Edinburgh Mental Well-being scale (WEMWBS) in the UK veterinary profession: Rasch analysis. Qual Life Res. 2013;22(2):379–91. 10.1007/s11136-012-0144-4. 22383106 10.1007/s11136-012-0144-4

[54] Hanzlova R, Lynn P. Item response theory-based psychometric analysis of the short Warwick-Edinburgh Mental Well-Being Scale (SWEMWBS) among adolescents in the UK. Health Qual Life Outcomes. 2023;21(1):108. 10.1186/s12955-023-02192-0. 37773069 10.1186/s12955-023-02192-0 PMC10540427

[55] Kraiss JT, Kohlhoff M, Ten Klooster PM. Disentangling between- and within-person associations of psychological distress and mental well-being: an experience sampling study examining the dual continua model of mental health among university students. Curr Psychol. 2023;4216789–16800. 10.1007/s12144-022-02942-1. 10.1007/s12144-022-02942-1 PMC8885315 35250243

