# Supplementary material for: The Short Warwick-Edinburgh Mental Well-being Scale (SWEMWBS): an Italian validation using confirmatory factor analysis and Rasch analysis
Source: BMC Psychol. 2024 Nov 21;12:680. doi: 10.1186/s40359-024-02177-8 (PMC11580197; doi:10.1186/s40359-024-02177-8)
Supplement: Supplementary file 1 — Supplementary Material 1. [file 40359_2024_2177_MOESM1_ESM.docx]

**Supplementary Materials**

**Title:** The Short Warwick-Edinburgh Mental Well-being Scale (SWEMWBS): An Italian validation using confirmatory factor analysis and Rasch analysis

**Table S1.** Descriptive statistics of the sample - Gender

| **Gender** | | **Frequency** | | **Percentage** | |  |
| --- | --- | --- | --- | --- | --- | --- |
| Female |  | 629 |  | 84.771 |  |  |
| Male |  | 113 |  | 15.229 |  |  |
| Total |  | 742 |  | 100.00 |  |  |

**Table S2.** Descriptive statistics of the sample – Civil Status

| **Civil status** | | **Frequency** | | **Percentage** | |  |
| --- | --- | --- | --- | --- | --- | --- |
| Divorced/Widowed |  | 32 |  | 4.313 |  |  |
| Engaged |  | 228 |  | 30.323 |  |  |
| Single |  | 232 |  | 30.323 |  |  |
| Married/cohabiting |  | 250 |  | 32.480 |  |  |
| Total |  | 742 |  | 100.00 |  |  |

**Table S3.** Descriptive statistics of the sample – Employed/study situation.

| **Job/study situation** | | **Frequency** | | **Percentage** | |  |
| --- | --- | --- | --- | --- | --- | --- |
| I am not employed and/or studying |  | 8 |  | 1.10 |  |  |
| Employed |  | 127 |  | 17.30 |  |  |
| Student |  | 171 |  | 23.90 |  |  |
| Student and employed |  | 436 |  | 57.50 |  |  |
| Total |  | 742 |  | 100.00 |  |  |

| **Table S4.** Descriptive statistics of the main variables | | | | | | | | | | | | | | | | | | | | | |
| --- | --- | --- | --- | --- | --- | --- | --- | --- | --- | --- | --- | --- | --- | --- | --- | --- | --- | --- | --- | --- | --- |
|  | **Mean** | | | **SD** | | **IQR** | | **Skewness** | | **SE** | | **Kurtosis** | | **SE** | | **25th percentile** | | **50th percentile** | | **75th percentile** | |
| SWEMWBS |  | 24.772 |  | 5.448 |  | 7.000 |  | -0.598 |  | 0.090 |  | 0.408 |  | 0.181 |  | 21.000 |  | 25.000 |  | 28.000 |  |
| WEMWBS |  | 42.065 |  | 9.478 |  | 12.000 |  | -0.538 |  | 0.091 |  | 0.199 |  | 0.182 |  | 36.000 |  | 43.000 |  | 48.000 |  |
| SWL |  | 22.583 |  | 6.834 |  | 10.000 |  | -0.276 |  | 0.090 |  | -0.600 |  | 0.180 |  | 18.000 |  | 23.000 |  | 28.000 |  |
| Stress |  | 11.140 |  | 5.400 |  | 8.000 |  | -0.055 |  | 0.091 |  | -0.761 |  | 0.181 |  | 7.000 |  | 11.000 |  | 15.000 |  |
| Anxiety |  | 7.078 |  | 5.751 |  | 9.000 |  | 0.614 |  | 0.091 |  | -0.694 |  | 0.182 |  | 2.000 |  | 6.000 |  | 11.000 |  |
| Depression |  | 7.937 |  | 5.987 |  | 10.000 |  | 0.485 |  | 0.091 |  | -0.866 |  | 0.181 |  | 3.000 |  | 7.000 |  | 13.000 |  |
| General Distress |  | 26.099 |  | 15.719 |  | 25.000 |  | 0.380 |  | 0.092 |  | -0.743 |  | 0.184 |  | 13.000 |  | 24.000 |  | 38.000 |  |
|  | | | | | | | | | | | | | | | | | | | | | |

Note. SWEMWBS= Short Warwick-Edinburgh Mental Well-being Scale, WEMWBS= Warwick-Edinburgh Mental Well-Being Scale

| **Table S5.** Descriptive statistics of the items in the SWEMWBS | | | | | | | | | | | | | | | | | | | | | | | |
| --- | --- | --- | --- | --- | --- | --- | --- | --- | --- | --- | --- | --- | --- | --- | --- | --- | --- | --- | --- | --- | --- | --- | --- |
|  | | **Mean** | | **SD** | | **Skewness** | | **SE** | | **Kurtosis** | | **SE** | | **Shapiro-Wilk** | | ***p*-value of Shapiro-Wilk** | | **25th percentile** | | **50th percentile** | | **75th percentile** | |
| SWEMWBS1 |  | 3.470 |  | 1.045 |  | -0.585 |  | 0.090 |  | -0.158 |  | 0.179 |  | 0.884 |  | < .001 |  | 3.000 |  | 4.000 |  | 4.000 |  |
| SWEMWBS2 |  | 3.623 |  | 1.033 |  | -0.674 |  | 0.090 |  | 0.006 |  | 0.179 |  | 0.875 |  | < .001 |  | 3.000 |  | 4.000 |  | 4.000 |  |
| SWEMWBS3 |  | 3.013 |  | 1.021 |  | -0.218 |  | 0.090 |  | -0.397 |  | 0.179 |  | 0.905 |  | < .001 |  | 2.000 |  | 3.000 |  | 4.000 |  |
| SWEMWBS4 |  | 3.505 |  | 0.985 |  | -0.486 |  | 0.090 |  | -0.073 |  | 0.179 |  | 0.889 |  | < .001 |  | 3.000 |  | 4.000 |  | 4.000 |  |
| SWEMWBS5 |  | 3.588 |  | 0.966 |  | -0.571 |  | 0.090 |  | 0.197 |  | 0.179 |  | 0.880 |  | < .001 |  | 3.000 |  | 4.000 |  | 4.000 |  |
| SWEMWBS6 |  | 3.746 |  | 1.039 |  | -0.740 |  | 0.090 |  | 0.089 |  | 0.179 |  | 0.867 |  | < .001 |  | 3.000 |  | 4.000 |  | 4.000 |  |
| SWEMWBS7 |  | 3.818 |  | 0.945 |  | -0.737 |  | 0.090 |  | 0.414 |  | 0.179 |  | 0.860 |  | < .001 |  | 3.000 |  | 4.000 |  | 4.000 |  |
|  | | | | | | | | | | | | | | | | | | | | | | | |

Note. SWEMWBS= Short Warwick-Edinburgh Mental Well-being Scale.

| **Table S6.** Spearman's correlations of the main variables | | | | | | | | | | | | | | | | | |
| --- | --- | --- | --- | --- | --- | --- | --- | --- | --- | --- | --- | --- | --- | --- | --- | --- | --- |
| **Variable** | |  | | **1** | | **2** | | **3** | | **4** | | **5** | | **6** | | **7** | |
| 1. SWEMWBS |  |  |  | — |  |  |  |  |  |  |  |  |  |  |  |  |  |
|  |  | Upper 95% CI |  | — |  |  |  |  |  |  |  |  |  |  |  |  |  |
|  |  | Lower 95% CI |  | — |  |  |  |  |  |  |  |  |  |  |  |  |  |
| 2. WEMWBS |  |  |  | 0.972 | *** | — |  |  |  |  |  |  |  |  |  |  |  |
|  |  | Upper 95% CI |  | 0.975 |  | — |  |  |  |  |  |  |  |  |  |  |  |
|  |  | Lower 95% CI |  | 0.967 |  | — |  |  |  |  |  |  |  |  |  |  |  |
| 3. SWL |  |  |  | 0.633 | *** | 0.631 | *** | — |  |  |  |  |  |  |  |  |  |
|  |  | Upper 95% CI |  | 0.674 |  | 0.673 |  | — |  |  |  |  |  |  |  |  |  |
|  |  | Lower 95% CI |  | 0.587 |  | 0.585 |  | — |  |  |  |  |  |  |  |  |  |
| 4. General Distress (DASS-21) |  |  |  | -0.535 | *** | -0.530 | *** | -0.484 | *** | — |  |  |  |  |  |  |  |
|  |  | Upper 95% CI |  | -0.480 |  | -0.474 |  | -0.426 |  | — |  |  |  |  |  |  |  |
|  |  | Lower 95% CI |  | -0.586 |  | -0.581 |  | -0.539 |  | — |  |  |  |  |  |  |  |
| 5. Stress (DASS-21) |  |  |  | -0.472 | *** | -0.462 | *** | -0.414 | *** | 0.931 | *** | — |  |  |  |  |  |
|  |  | Upper 95% CI |  | -0.413 |  | -0.402 |  | -0.352 |  | 0.940 |  | — |  |  |  |  |  |
|  |  | Lower 95% CI |  | -0.527 |  | -0.518 |  | -0.472 |  | 0.921 |  | — |  |  |  |  |  |
| 6. Anxiety (DASS-21) |  |  |  | -0.417 | *** | -0.417 | *** | -0.373 | *** | 0.900 | *** | 0.775 | *** | — |  |  |  |
|  |  | Upper 95% CI |  | -0.354 |  | -0.354 |  | -0.308 |  | 0.913 |  | 0.803 |  | — |  |  |  |
|  |  | Lower 95% CI |  | -0.476 |  | -0.477 |  | -0.434 |  | 0.885 |  | 0.744 |  | — |  |  |  |
| 7. Depression (DASS-21) |  |  |  | -0.564 | *** | -0.561 | *** | -0.534 | *** | 0.917 | *** | 0.788 | *** | 0.730 | *** | — |  |
|  |  | Upper 95% CI |  | -0.512 |  | -0.508 |  | -0.480 |  | 0.928 |  | 0.814 |  | 0.763 |  | — |  |
|  |  | Lower 95% CI |  | -0.612 |  | -0.609 |  | -0.584 |  | 0.904 |  | 0.759 |  | 0.694 |  | — |  |
|  | | | | | | | | | | | | | | | | | |
| Note. *p* < .01, *** *p* < .001. SWEMWBS= Short Warwick-Edinburgh Mental Well-being Scale, WEMWBS= Warwick-Edinburgh Mental Well-Being Scale, SWL= Satisfaction With Life Scale | | | | | | | | | | | | | | | | | |

**The following results are extracted directly from the R code using the” measureQ” package:**

**Model code:**

Model.A <- '

WB =~ SWEMWBS1+SWEMWBS2+SWEMWBS3+SWEMWBS4+SWEMWBS5+ SWEMWBS6+SWEMWBS7

DEP =~ DASS3+DASS5+DASS10+DASS13+DASS16+DASS17+DASS21

ANX =~ DASS2+DASS4+DASS7+DASS9+DASS15+DASS19+DASS20

STS =~ DASS1+DASS6+DASS8+DASS11+DASS12+DASS14+DASS18

LS =~ SWLS1+SWLS2+SWLS3+SWLS4+SWLS5’

| **Table S7.** Standardized Factor Loadings | | |  |  |
| --- | --- | --- | --- | --- |
|  |  |  |  |  |
| Factor Loading | Estimate |  |  |  |
| **WB =~ SWEMWBS1** | **0.804** |  |  |  |
| **WB =~ SWEMWBS2** | **0.662** |  |  |  |
| **WB =~ SWEMWBS3** | **0.708** |  |  |  |
| **WB =~ SWEMWBS4** | **0.793** |  |  |  |
| **WB =~ SWEMWBS5** | **0.837** |  |  |  |
| **WB =~ SWEMWBS6** | **0.734** |  |  |  |
| **WB =~ SWEMWBS7** | **0.839** |  |  |  |
| DEP =~ DASS3 | 0.737 |  |  |  |
| DEP =~ DASS5 | 0.661^c^ |  |  |  |
| DEP =~ DASS10 | 0.835 |  |  |  |
| DEP =~ DASS13 | 0.857 |  |  |  |
| DEP =~ DASS16 | 0.801 |  |  |  |
| DEP =~ DASS17 | 0.828 |  |  |  |
| DEP =~ DASS21 | 0.797 |  |  |  |
| ANX =~ DASS2 | 0.512^c^ |  |  |  |
| ANX =~ DASS4 | 0.822 |  |  |  |
| ANX =~ DASS7 | 0.712 |  |  |  |
| ANX =~ DASS9 | 0.776 |  |  |  |
| ANX =~ DASS15 | 0.852 |  |  |  |
| ANX =~ DASS19 | 0.794 |  |  |  |
| ANX =~ DASS20 | 0.752 |  |  |  |
| STS =~ DASS1 | 0.737 |  |  |  |
| STS =~ DASS6 | 0.713 |  |  |  |
| STS =~ DASS8 | 0.793 |  |  |  |
| STS =~ DASS11 | 0.806 |  |  |  |
| STS =~ DASS12 | 0.811 |  |  |  |
| STS =~ DASS14 | 0.737 |  |  |  |
| STS =~ DASS18 | 0.776 |  |  |  |
| LS =~ SWLS1 | 0.803 |  |  |  |
| LS =~ SWLS2 | 0.818 |  |  |  |
| LS =~ SWLS3 | 0.908 |  |  |  |
| LS =~ SWLS4 | 0.733 |  |  |  |
| LS =~ SWLS5 | 0.704 |  |  |  |
|  |  |  |  |  |
| Note:   \| c = standardized factor loading significantly less than 0.7 (*p* < .05) \| \| --- \|   WB= Well Belling, DEP= Depression, ANX= Anxiety, STS= Stress, LS= Life satisfaction | | | | |
|  | | | | |

| **Table S8.** Descriptive statistics (Observed Mean, Latent SD, AVE, Construct Reliability, Latent Correlation) | | | | | | | |  |  |
| --- | --- | --- | --- | --- | --- | --- | --- | --- | --- |
|  |  |  |  |  |  |  |  |  |  |
| **Factor** | **Mean** | **SD** | **AVE** | **1** | **2** | **3** | **4** | **5** |  |
| **1.WB** | **3.427** | **0.832** | **0.520** | **(0.881)** |  |  |  |  |  |
| 2.DEP | 1.143 | 0.713 | 0.625 | -0.623 | (0.921) |  |  |  |  |
| 3.ANX | 1.019 | 0.492 | 0.567 | -0.460 | 0.779c | (0.900) |  |  |  |
| 4.STS | 1.593 | 0.661 | 0.591 | -0.531 | 0.847#b | 0.831#b | (0.910) |  |  |
| 5. LS | 4.491 | 1.239 | 0.634 | 0.728 | -0.603 | -0.430 | -0.468 | (0.896) |  |
|  |  |  |  |  |  |  |  |  |  |
| Note: AVE = Average Variance Extracted; | | | | | | |  |  |  |
| diagonal elements in brackets = Construct Reliability | | | | |  |  |  |  |  |
| B = Construct Reliability significantly lower than 0.8 (*p* <0.05) | | | | | | | | |  |
| Correlation coefficient: c = significantly larger than 0.7 (*p* <0.05) | | | | | | | | | |
| # = AVE is significantly less than squared-correlation (*p* < 0.05) | | | | | |  |  |  |  |

WB= Well Belling, DEP= Depression, ANX= Anxiety, STS= Stress, LS= Life satisfaction

| **Table S9.** Descriptive statistics (Observed Mean, Observed SD, AVE, Reliability, Observed Correlation) | | | | | | | |  |  |
| --- | --- | --- | --- | --- | --- | --- | --- | --- | --- |
|  |  |  |  |  |  |  |  |  |  |
| **Factor** | **Mean** | **S.D.** | **AVE** | **1** | **2** | **3** | **4** | **5** |  |
| **1.WB** | **3.427** | **0.793** | **0.520** | **(0.877)** |  |  |  |  |  |
| 2.DEP | 1.143 | 0.854 | 0.625 | -0.555 | (0.919) |  |  |  |  |
| 3.ANX | 1.019 | 0.820 | 0.567 | -0.408 | 0.729 | (0.897) |  |  |  |
| 4.STS | 1.593 | 0.771 | 0.591 | -0.467 | 0.788 | 0.769 | (0.908) |  |  |
| 5.LS | 4.491 | 1.360 | 0.634 | 0.640 | -0.543 | -0.384 | -0.419 | (0.889) |  |
|  |  |  |  |  |  |  |  |  |  |
| Note: AVE = Average Variance Extracted. WB= Well Belling, DEP= Depression, ANX= Anxiety, STS= Stress, LS= Life satisfaction. Diagonal elements in brackets = Cronbach's Alpha. | | | | | | |  |  |  |
|  | | | | |  |  |  |  |  |

| **Table S10.** Heterotrait-monotrait ratio | | | | | | | | | |
| --- | --- | --- | --- | --- | --- | --- | --- | --- | --- |
| **SWEMWBS** | | **SWL** | | **Stress** | | **Anxiety** | | **Depression** | |
| **0.718** |  | - |  |  |  |  |  |  |  |
| **0.527** |  | 0.482 |  | - |  |  |  |  |  |
| **0.471** |  | 0.447 |  | 0.834 |  | - |  |  |  |
| **0.644** |  | 0.622 |  | 0.819 |  | 0.807 |  | - |  |
|  | | | | | | | | | |

Note: SWEMWBS= Short Warwick-Edinburgh Mental Well-being Scale, SWL = Satisfaction With Life Scale

| **Table S11.** Factor loadings of items in the Short Warwick-Edinburgh Mental Well-being Scale | | | | | | | | | | | | | | | | | |
| --- | --- | --- | --- | --- | --- | --- | --- | --- | --- | --- | --- | --- | --- | --- | --- | --- | --- |
|  | | | | | | | | | | | | 95% Confidence Interval | | | |  | |
| Factor | | Indicator | | Estimate | | Std. Error | | z-value | | *p* | | Lower | | Upper | | **Std. Est. (all)** | |
| WEMWBS |  | WEMWBS1 |  | 0.827 |  | 0.032 |  | 25.549 |  | < .001 |  | 0.763 |  | 0.890 |  | 0.792 |  |
|  |  | WEMWBS2 |  | 0.681 |  | 0.034 |  | 19.768 |  | < .001 |  | 0.613 |  | 0.748 |  | 0.658 |  |
|  |  | WEMWBS3 |  | 0.721 |  | 0.033 |  | 21.610 |  | < .001 |  | 0.655 |  | 0.786 |  | 0.705 |  |
|  |  | WEMWBS4 |  | 0.548 |  | 0.038 |  | 14.490 |  | < .001 |  | 0.474 |  | 0.622 |  | 0.512 |  |
|  |  | WEMWBS5 |  | 0.955 |  | 0.037 |  | 26.148 |  | < .001 |  | 0.883 |  | 1.026 |  | 0.805 |  |
|  |  | WEMWBS6 |  | 0.822 |  | 0.030 |  | 27.550 |  | < .001 |  | 0.764 |  | 0.881 |  | 0.833 |  |
|  |  | WEMWBS7 |  | 0.729 |  | 0.031 |  | 23.720 |  | < .001 |  | 0.668 |  | 0.789 |  | 0.754 |  |
|  |  | WEMWBS8 |  | 0.639 |  | 0.035 |  | 18.134 |  | < .001 |  | 0.570 |  | 0.708 |  | 0.616 |  |
|  |  | WEMWBS9 |  | 0.930 |  | 0.033 |  | 27.988 |  | < .001 |  | 0.865 |  | 0.996 |  | 0.842 |  |
|  |  | WEMWBS10 |  | 0.743 |  | 0.029 |  | 25.183 |  | < .001 |  | 0.685 |  | 0.800 |  | 0.785 |  |
|  |  | WEMWBS11 |  | 0.784 |  | 0.038 |  | 20.684 |  | < .001 |  | 0.710 |  | 0.859 |  | 0.681 |  |
|  |  | WEMWBS12 |  | 0.798 |  | 0.029 |  | 27.253 |  | < .001 |  | 0.741 |  | 0.855 |  | 0.827 |  |
|  | | | | | | | | | | | | | | | | | |

**Appendix**

**Short Warwick-Edinburgh Mental Well-Being Scale Italian version**

Scala likert - 1= Mai 5= Molto spesso

1= Mai, 2= Raramente, 3= Qualche volta, 4= Spesso, 5= Molto spesso

**Di seguito sono presenti delle affermazioni che fanno riferimento ai suoi pensieri e ai tuoi stati d'animo/sentimenti, in riferimento alle ultime due settimane. Non esistono risposte giuste o sbagliate, indica quale ti rappresenta meglio:**

1 Mi sono sentito/a ottimista riguardo al futuro

2 Mi sono sentito/a utile

3 Mi sono sentito/a rilassato

7 Ho affrontato bene i problemi

8 Ho pensato in modo chiaro

9 Mi sono sentito/a vicino ad altre persone

11 Sono stato/a in grado di prendere decisioni

Il punteggio totale varia da 7 a 35. Si sommano i 7 item. Un punteggio più alto indica un maggiore benessere mentale.
